# Torsion of epididymal Cyst as a cause of acute Scrotum in a child

**DOI:** 10.1016/j.eucr.2023.102417

**Published:** 2023-05-03

**Authors:** Mehdi Vafadar, Nima Rakhshankhah, Mahmoud Salek, Elham Zarei

**Affiliations:** a Pediatric Endocrinology Department, Ali Asghar Children Hospital, School of Medicine, Iran University of Medical Sciences, Tehran, Iran; b Department of Diagnostic Radiology, Iran University of Medical Science, Tehran, Iran; c Pediatric Surgery Department, Ali Asghar Children Hospital, School of Medicine, Iran University of Medical Sciences, Tehran, Iran; d Radiology Department, Ali Asghar Children Hospital, School of Medicine, Iran University of Medical Sciences (IUMS), Tehran, Iran

**Keywords:** Testicular torsion, Cyst, Epididymis, Acute scrotum

## Abstract

Epididymal cyst is commonly seen in adults and sometimes in children. In most cases of the epididymal cyst, there are no symptoms and the diagnosis is made incidentally during ultrasonography. However, in rare circumstances, such as infection, trauma, or torsion, an epididymal cyst could become painful and require surgical or medical intervention. We report the case of an 11-year-old boy admitted to our hospital with acute right scrotal pain and treated surgically for epidydimal cyst torsion. Torsed epididymal cysts may cause symptoms exactly like testicular torsion. Therefore, It should be considered a differential diagnosis of testicular torsion in pediatrics.

## 1 Introduction

Epididymal cysts (ECs) are benign cystic lesions that are common in adults in comparison to pediatrics.^1^^,^^2^ It is not known exactly how EC develops, although some literature imputes it to epidydimal/lymphatic duct obstruction and some of them prefer hormonal changes as the main cause.^3^ In most cases of EC, there are no symptoms or signs, and the diagnosis is made incidentally by ultrasound. EC can become painful and require surgical or medical treatment in rare conditions, such as infection, trauma, or torsion.^1^^,^^4^ EC torsion is very rare and to the best of our knowledge, only 9 cases have been reported in the literature.^4^

## 2 Case report

An 11-year-old boy was admitted to our hospital with acute pain in the right scrotum lasting 24 hours. There were no urological symptoms and no history of previous scrotal trauma. On physical examination tenderness of the right scrotum, swelling, and erythema was noted. Abdominal and groin examinations were unremarkable. All routine hematology, urinalysis, and serum biochemistry were normal.

**Fig. 1:**
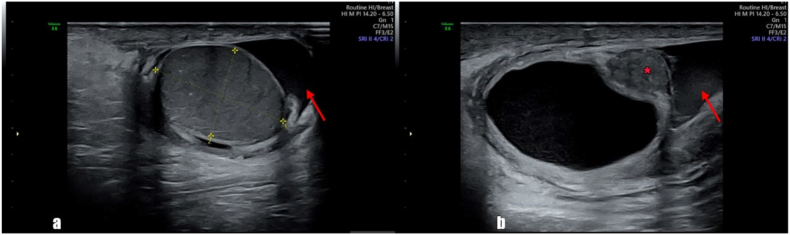
Ultrasonography (**a**) shows the normal appearance right testis with mild right hydrocele (arrow). Evidence of a thick-walled cyst with a heterogenous appearance epidydimal head(*) and mild right side hydrocele (arrow) is noted (**b**).

Ultrasonography showed normal size, echogenicity, and vascularity of both testes. The left epididymis showed normal appearance and vascularity. On the right side, a thick-walled cystic lesion measured 30 × 23 × 18 mm with internal echoes was seen in the head of the epididymis (Fig. 1). An increase in the size and heterogeneity of the right epididymis along with an increase in vascularity of the epididymis and twisted pedicle structure adjacent to the epididymal head was noted.

**Fig. 2:**
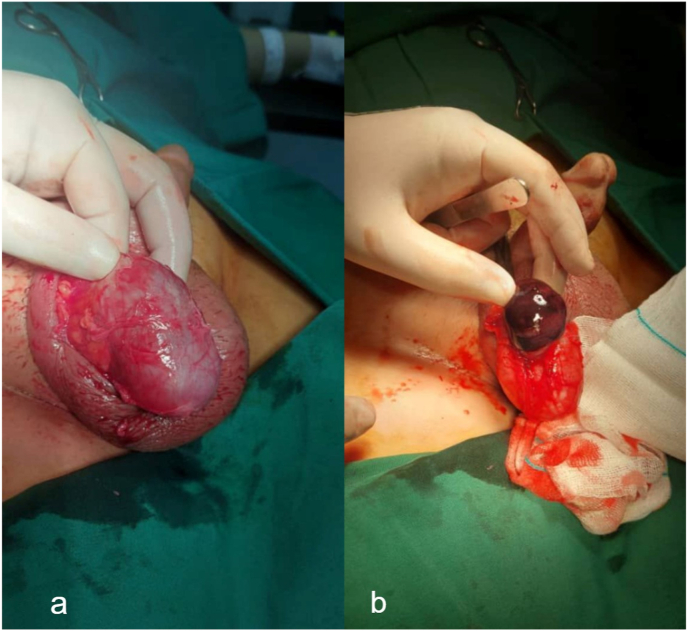
Normal appearance of right testis after the opening of tunica vaginalis(**a**). A torsed necrotic epididymal cyst is seen adjacent to the right epidydimal head (**b**).

The patient was admitted to the operating room with a diagnosis of epididymal cyst torsion. On the supine position under the transverse scrotal incision, the tunica vaginalis was opened. On surgical exploration, a large cyst was seen connected to the head of the epididymis by a twisted pedicle with torsion-developed necrosis (Fig. 2). Therefore removal of the cyst, drainage of the hydrocele, and closure of the tunica vaginalis were performed. Pathologic evaluation showed a mesothelial lined cystic lesion with hemorrhagic-necrotic components. The patient was discharged from the hospital 3 days after surgery in good physical condition.

## 3 Discussion

The acute scrotum is common in the pediatric population. Several etiologies should be considered for this setting, and almost all are best diagnosed with examination and ultrasonography.^2^ ECs are usually seen in the adult population, but can also occur in pediatrics.^1^ In most cases with EC conservative management has been suggested, but in the setting of acute scrotum further evaluation is required.^4^ Color Doppler ultrasonography is the modality of choice for differentiating the causes of the acute scrotum from one another.^2^ As far as we know, only nine cases have been reported in Literature.^4^ It is the third case of a right-sided EC with acute scrotum presentation^1^^,^^4^ and the remaining previous seven cases are left-sided lesions.^3^^,^^5^ Most of the torsed EC patients are older than 10-year-old boys.^4^ And almost all of them show acute scrotum symptoms when becoming larger than 30 mm; like in our case.^5^ In our case, surgical excision was done due to intractable scrotal pain, and confirmed ultrasound findings similar to previous cases.

In conclusion although the clinical symptoms of torsed ECs are exactly similar to testicular torsion, due to the non-emergency nature of torsed ECs surgery, correct diagnosis with ultrasound is very important.

## Funding

No funding was obtained for this study.

## Authors’ contributions

NR, MV, MS, and EZ participated in writing and helped to draft the manuscript. All authors read and approved the final manuscript.

## Data availability

All relevant data are shown in the manuscript.

## Ethics approval and consent to participate

The patient gave written informed consent to participate.

## Declaration of competing interest

The authors declare that they have no known competing financial interests or personal relationships that could have appeared to influence the work reported in this paper.
